# Supplementary material for: A clinically applicable deep-learning model for detecting intracranial aneurysm in computed tomography angiography images
Source: Nat Commun. 2020 Nov 30;11:6090. doi: 10.1038/s41467-020-19527-w (PMC7705757; doi:10.1038/s41467-020-19527-w)
Supplement: Supplementary file 1 — Supplementary Information [file 41467_2020_19527_MOESM1_ESM.docx]

**Title: A Clinically Applicable Deep Learning Model for Detecting Intracranial Aneurysm in Computed Tomography Angiography Images**

**Supplementary Materials**

Supplementary Figures and Figure Legends

**
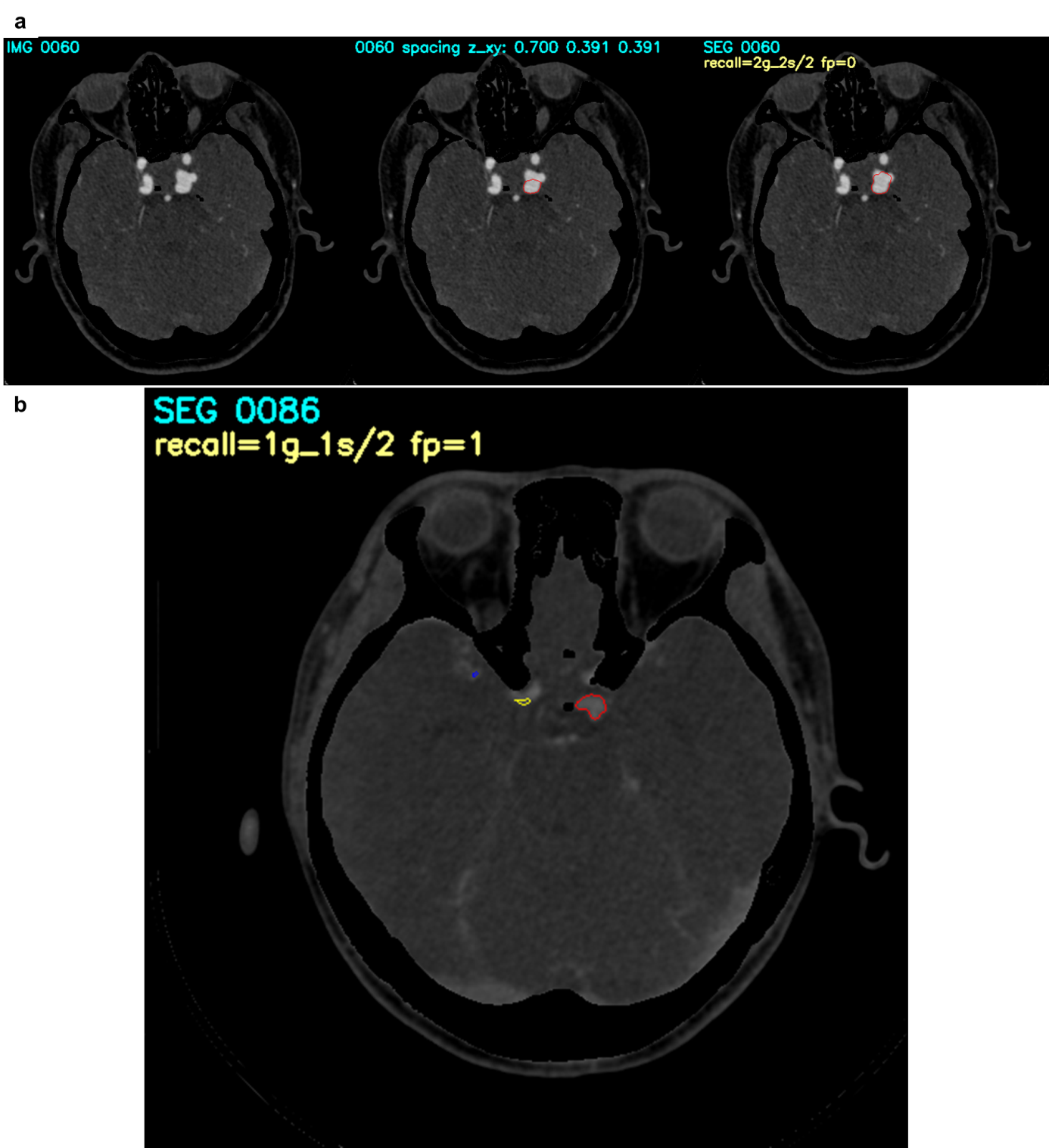
**

Supplementary Fig. 1 | Sketch map of the row image, ground truth and the prediction result. **a** This is the segmentation-based method used to achieve the detection task. The first picture is a cross-sectional digital subtraction bone-removal CT image in a patient with left middle cerebral artery aneurysm. The middle picture shows the segmented result (red circle). The third picture shows how the predicted results are presented. **b** Results of the model prediction. The red outline indicates the correctly predicted aneurysms according to the ground truth. The yellow marker indicates the missed ground truth, and the blue indicates false negative predictions.


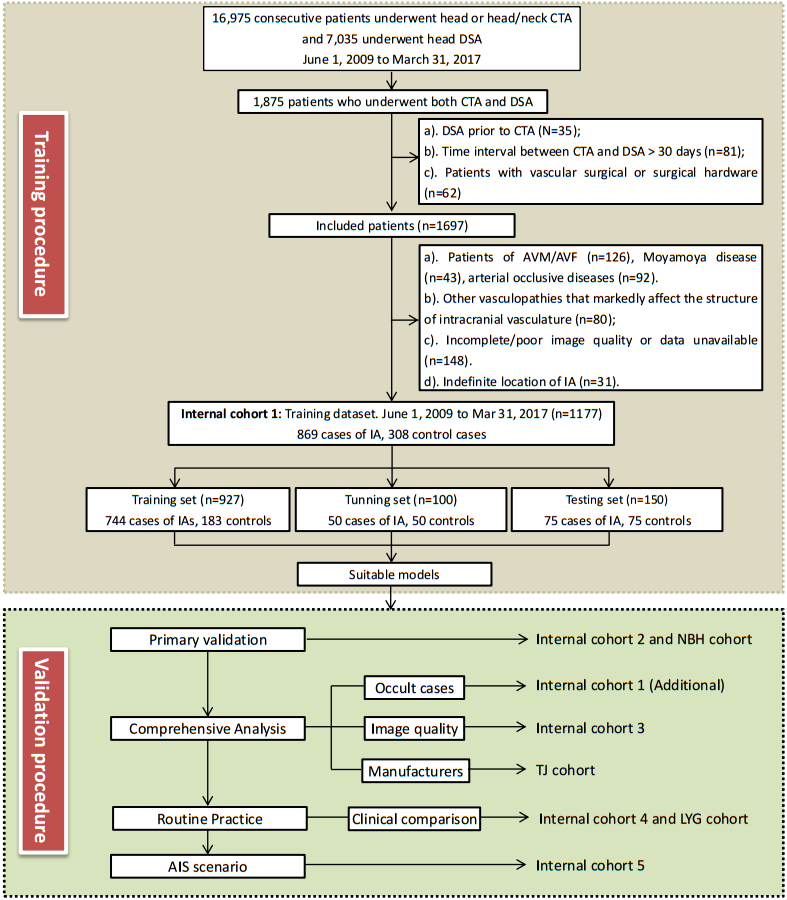


Supplementary Fig. 2 | Flowchart of the training and validation procedures of our proposed model. AIS, acute ischemic stroke; AVM/AVF, arteriovenous malformation / fistula; CTA, Computed tomography angiography; DSA, digital subtraction angiography; IA, intracranial aneurysm. NBH cohort, Nanjing Brain Hospital cohort; TJ cohort, Tianjin First Central Hospital cohort; LYG cohort, Lianyungang First People’s Hospital.

Note: 31 occult cases were found in Internal cohort 1.


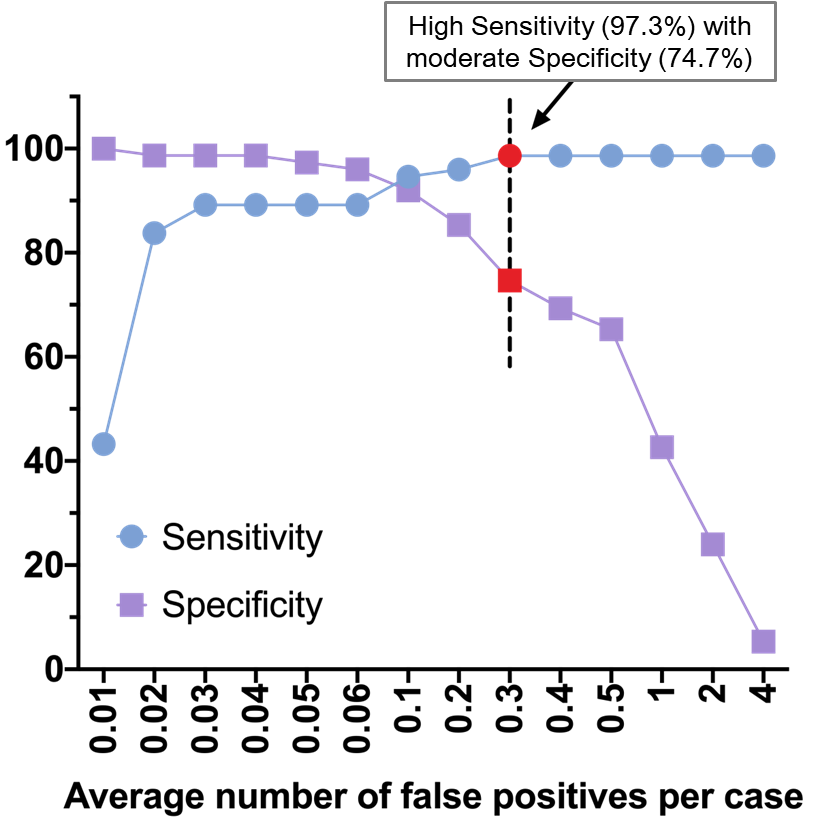


Supplementary Fig. 3 | Performance of models with different false positives (FPs). The model achieved high patient-level sensitivity of 97.3% and moderate specificity of 74.7% when FP number per CTA scan equals to 0.29.


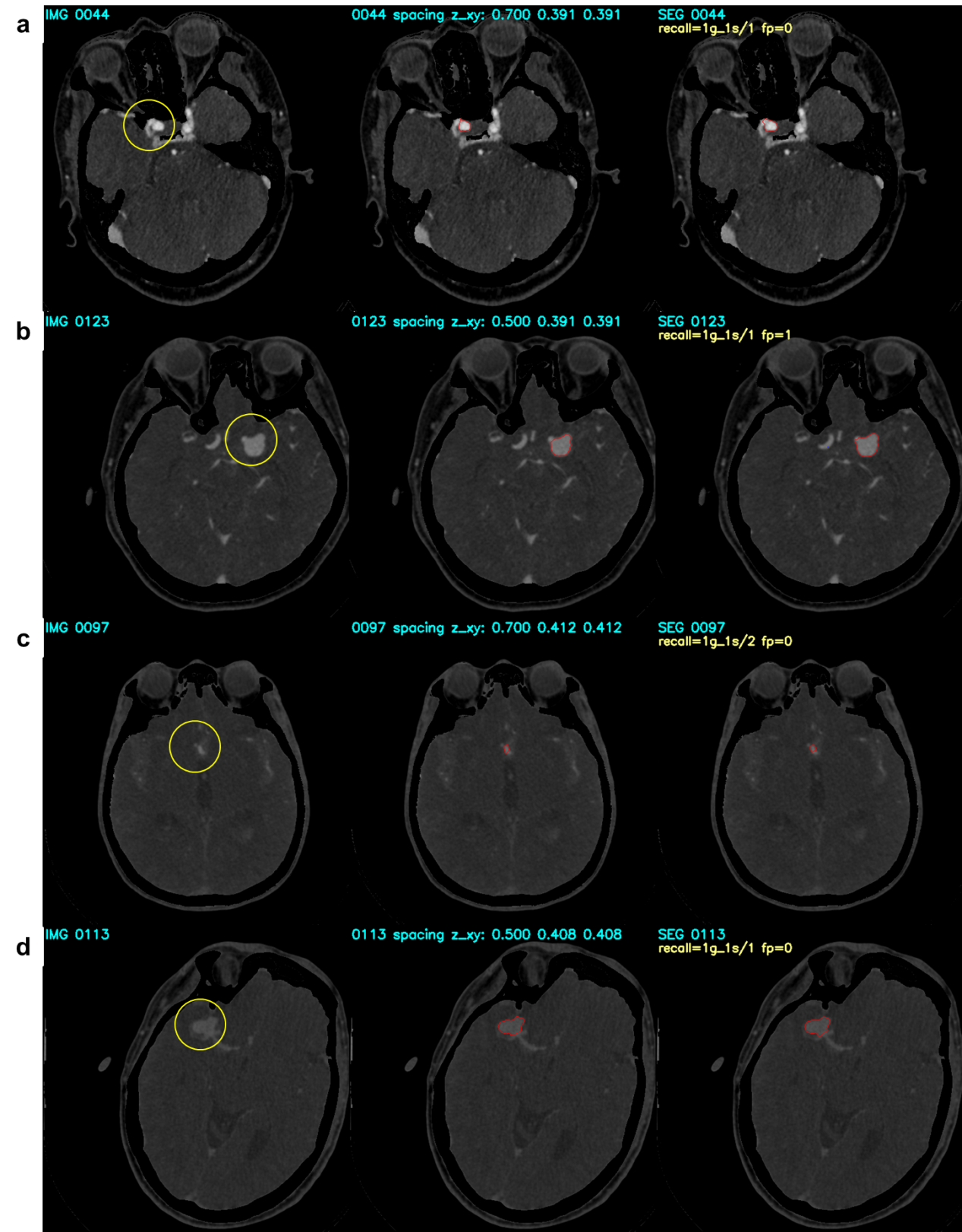


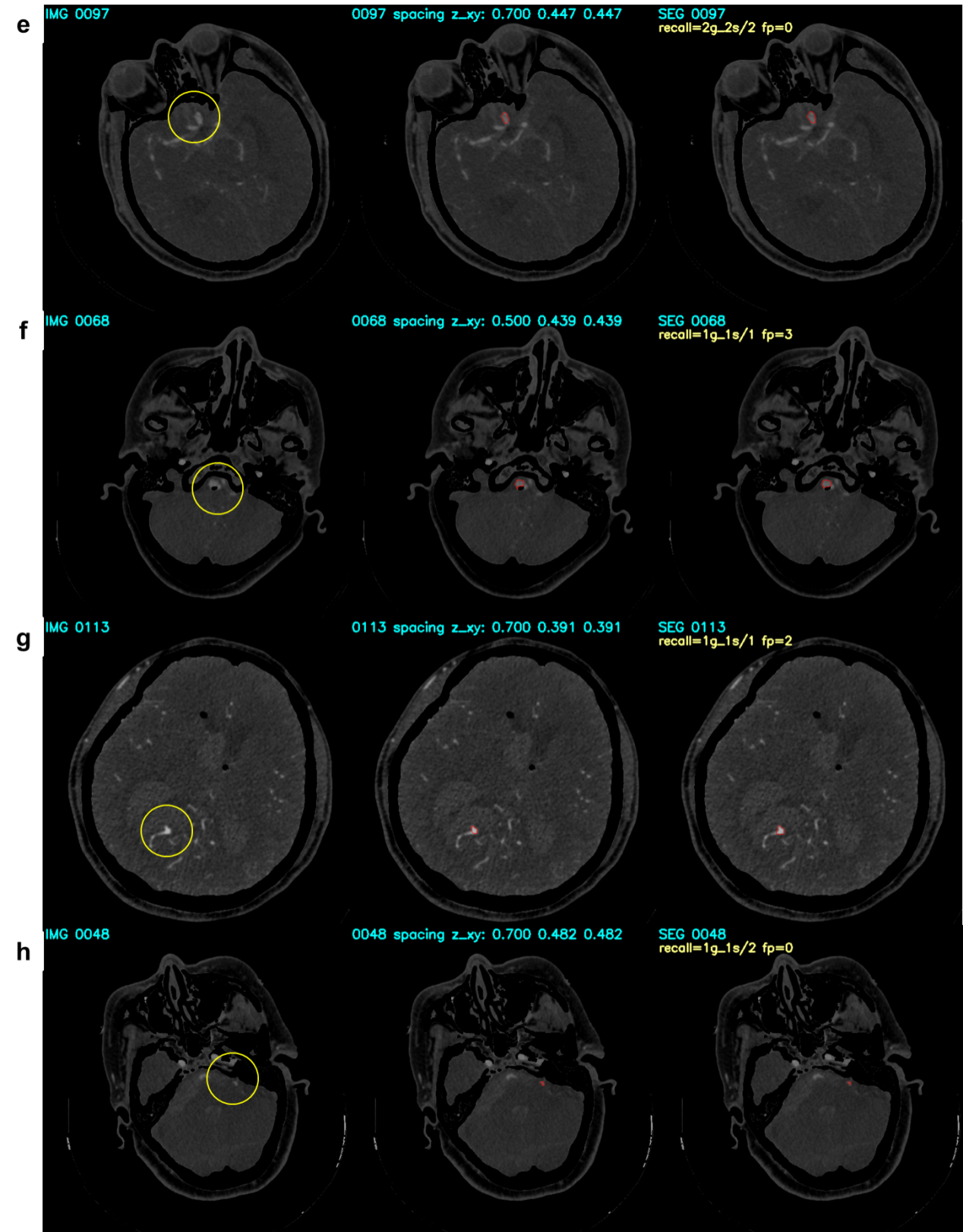


Supplementary Fig. 4 | Samples of the correctly diagnosed cases in different locations. The yellow circles indicate the location of aneurysms. In each panel of a-h, the first picture is the raw cross-sectional digital subtraction bone-removal CT image, the middle picture shows the segmented result (red outline), and the third picture shows the predicted results by the model (red outline).


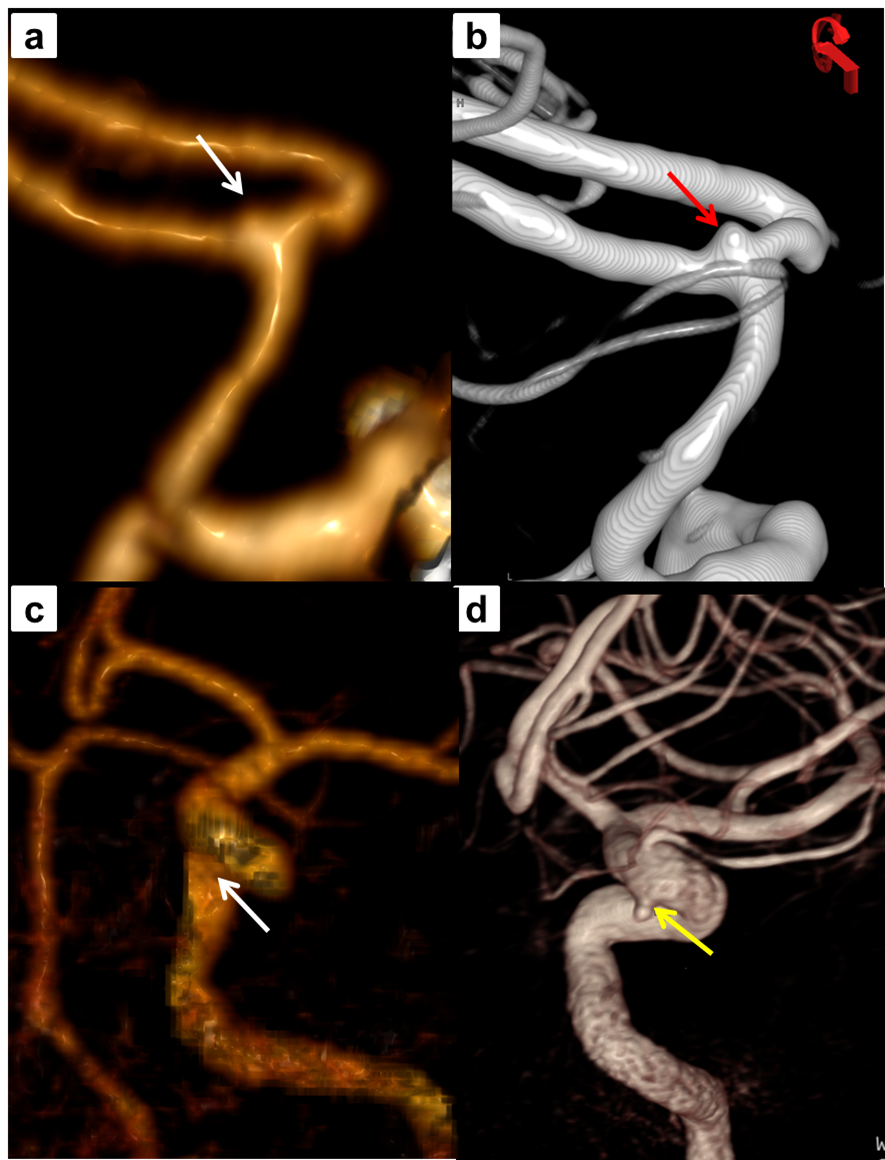


Supplementary Fig. 5 | Examples of occult cases of CTA-negative but DSA-positive aneurysms. **a** Volume-rendered CT angiography image of an aneurysm of the anterior communication artery. **b** Volume-rendered 3D DSA image clearly shows the aneurysm from the same case (red arrow). **c** Volume-rendered CT angiography image does not clearly present the right internal carotid artery aneurysm (white arrow). **d** Volume-rendered 3D DSA image clearly shows the aneurysm (yellow arrow).


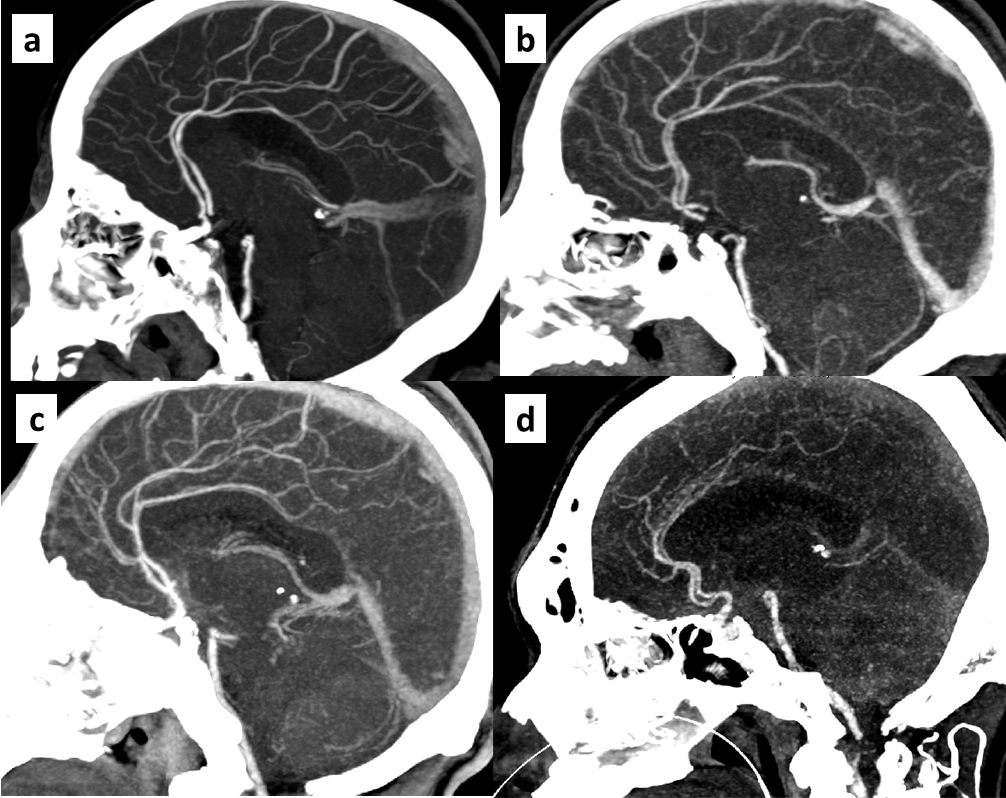


Supplementary Fig. 6 | Examples of cerebral CTA image quality on 4-point scale for overall subjective evaluation. **a** sagittal maximum intensity projection (MIP) reformatted image shows image quality of score 4 with little noise and the sharpest vessel contour; **b** sagittal MIP reformatted image shows image quality of score 3; **c** sagittal MIP reformatted image shows image quality of score 2; **d** sagittal MIP reformatted image shows image quality of score 1, in which intracranial arteries are too noisy and blurry to diagnose.


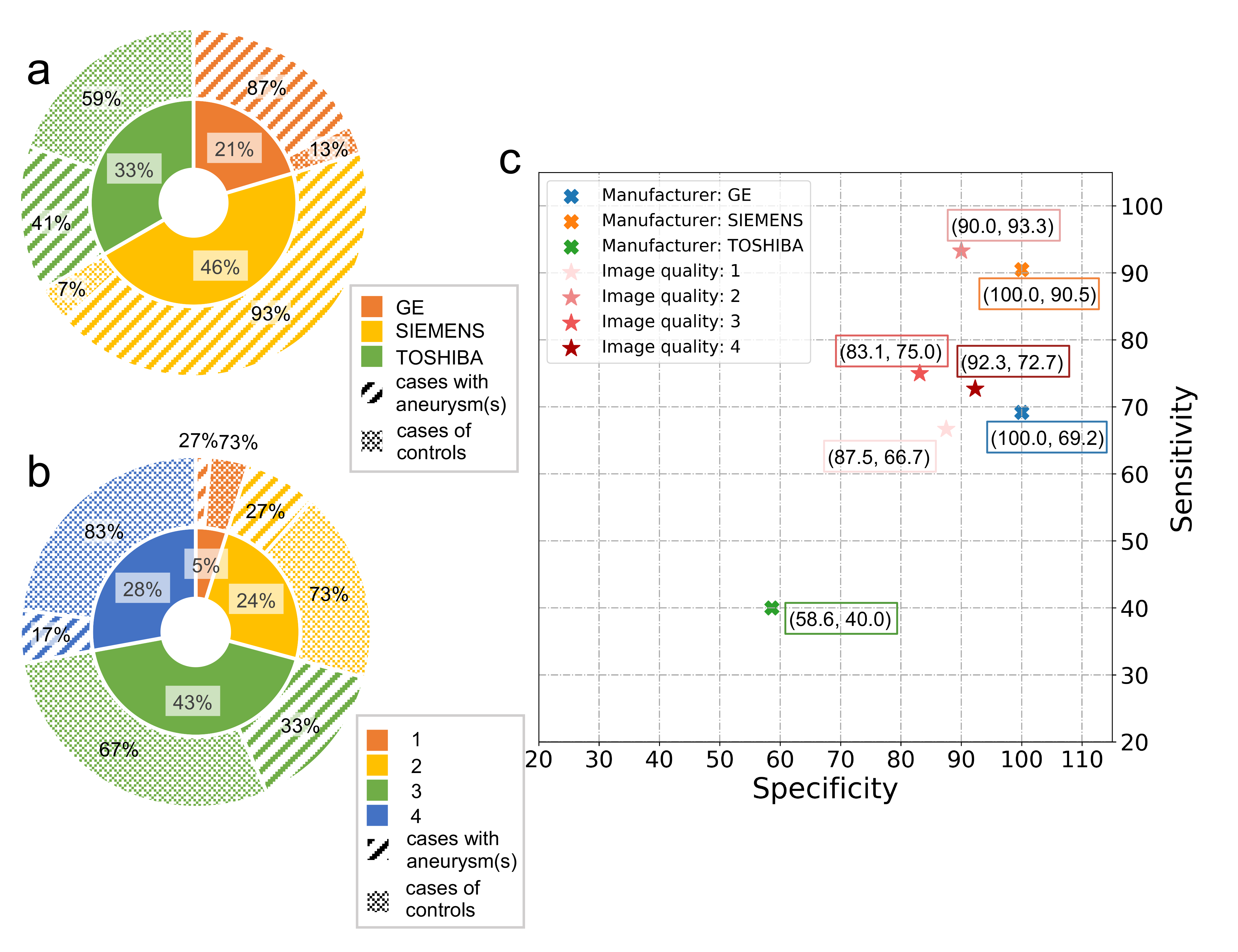


Supplementary Fig. 7 | Influence of image quality and manufacturers on model performance. **a** The distribution of the image quality with score 1-4 and the proportion of cases with aneurysm(s). **b** The distribution of the 3 manufacturers (GE, SIEMENS, and TOSHIBA) and the proportion of cases with aneurysm(s). **c** Corresponding diagnostic performance of the model with different manufacturers (Bonferroni-correction was conducted and *p*<0.017 was used as a statistical significance threshold. For sensitivity, *χ_c_*^2^=4.767, *p*=0.029 between SIEMENS and GE; *χ_c_*^2^=19.899, *p*<0.001 between SIEMENS and TOSHIBA; *χ*^2^=3.930, *p*=0.047 between GE and TOSHIBA. For specificity, Fisher exact test, *p*＞0.999 between SIEMENS and GE; Fisher exact test, *p*=0.137 between SIEMENS and TOSHIBA; Fisher exact test, *p*=0.271 between GE and TOSHIBA) and image qualities (Bonferroni-correction was conducted and *p*<0.008 was used as a statistical significance threshold. For sensitivity, Fisher exact test, *p*=0.271 between score 1 and 2; Fisher exact test, *p*＞0.999 between score 1 and 3, score 1 and 4; *χ_c_*^2^=1.191, *p*=0.275 between score 2 and 3; Fisher exact test, *p*=0.279 between score 2 and 4; *χ_c_*^2^=0.000, *p*＞0.999 between score 3 and 4. For specificity, *χ_c_*^2^=0.000, *p*＞0.999 between score 1 and 2, score 1 and 3, and score 1 and 4; *χ*^2^=0.969, *p*=0.325 between score 2 and 3; *χ*^2^=0.392, *p*=0.531 between score 2 and 4; *χ*^2^=0.158, *p*=0.691 between score 3 and 4), and no significant differences were found (all Bonferroni-corrected *p*>0.05) in patient-level sensitivity and specificity. Two-sided Pearson’s chi-squared test or Fisher exact test with Bonferroni-correction was conducted.


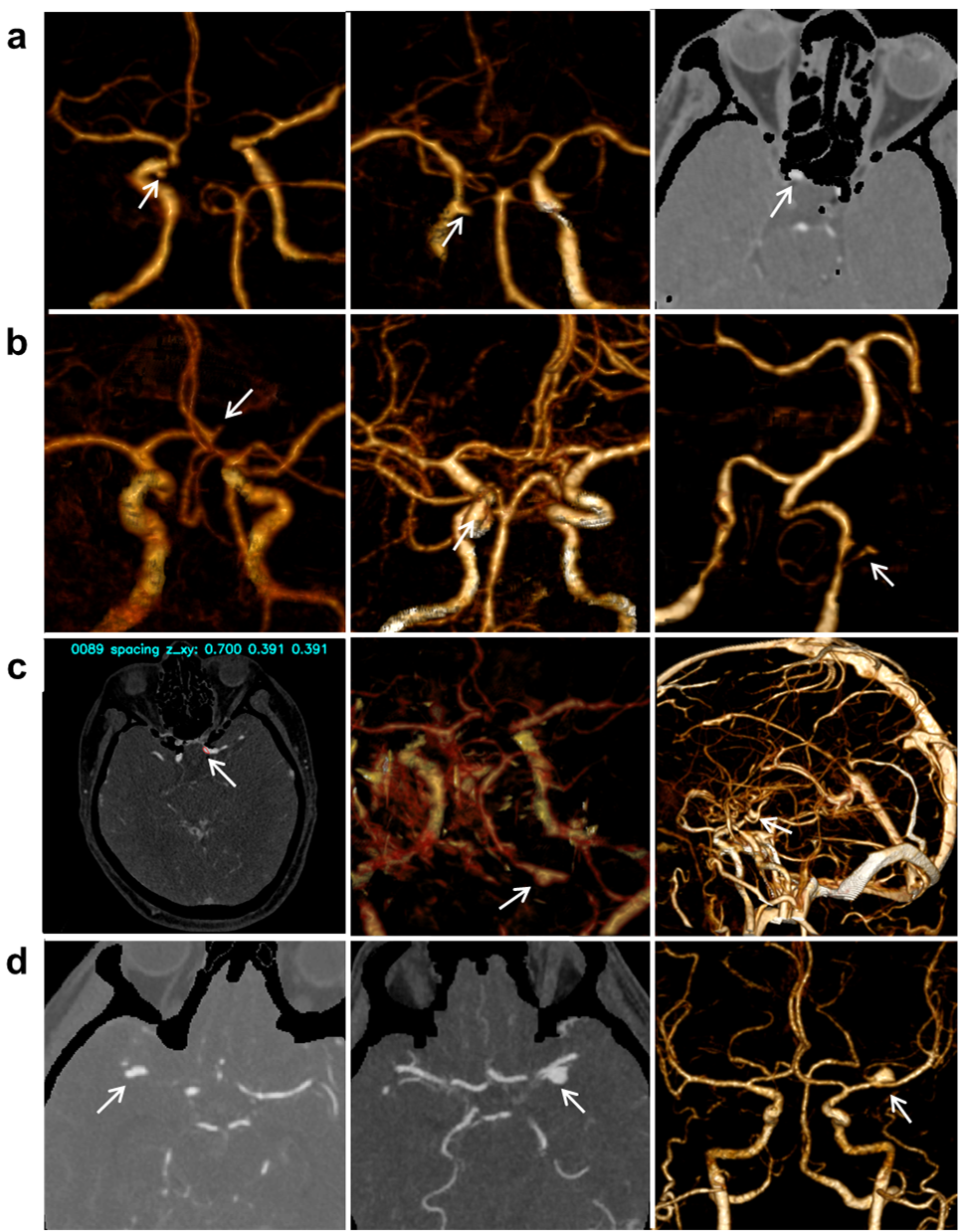


Supplementary Fig. 8 | Analysis of the classification errors on the test sets. **a** The left case shows a tiny aneurysm (1.5 mm in size, arrow) at the ophthalmic artery segment of the left internal carotid artery, which was missed by the model. The middle and right images (volume rendering [VR] and axial images) show one missed aneurysm of the right ophthalmic artery segment with 5.2 mm in size (arrow) which may be attributed to overshooting of bone subtraction. **b** Uncommon morphology and locations of aneurysms (arrows). The left VR image shows an aneurysm with strip-like morphology of the anterior communication artery (arrow). The middle one was a blood blister-like aneurysm at the supraclinoidal portion of the right internal carotid artery (arrow). The right VR image shows an aneurysm of the posterior inferior cerebellar artery (arrow), uncommon location for IAs. **c** This picture shows a rare blood blister-like aneurysm of the ophthalmic artery segment of the right internal carotid artery. The middle and right VR images show poor artery enhancement and marked intracranial vein enhancement because of inappropriate contrast agent protocols for diagnosis of an aneurysm at the right vertebral basilar artery and anterior choroidal artery of the left internal carotid artery (arrow), respectively. **d** Missed with other unexplained reasons. The 3 images show two easily recognizable aneurysms of the middle cerebral artery (arrows) of 7 mm in size acquired by two different CT scanners (GE Revolution and TOSHIBA Aquilion ONE).

Supplementary Table 1 | Overview of datasets used for training, validation and testing of the model.

| **Number** | **Cohort** | **Scanner type** | **Patients, n** | **Summary** | **Cases, n (%)** | |
| --- | --- | --- | --- | --- | --- | --- |
|  |  |  |  |  | **Cases with IA** | **Control** |
| #1 | **Internal cohort 1** | SOMATOM Definition and SOMATOM Definition Flash | 1177 | DSA-verified consecutive eligible CTA cases for model training from Jun. 1, 2009 to Mar. 31, 2017 in Jinling Hospital. | 869 (73.8) | 308 (26.2) |
| #2 | **Internal cohort 2** | SOMATOM Definition and SOMATOM Definition Flash | 245 | DSA-verified consecutive eligible CTA cases for internal validation from Apr. 1, 2017 to Dec. 31, 2017 in Jinling Hospital. | 108 (44.1) | 137 (55.9) |
| #3 | **Internal cohort 3** | SOMATOM Definition and SOMATOM Definition Flash | 226 | DSA-verified consecutive eligible CTA cases for validation of the effect of image quality from Jan.1, 2018 to May. 31, 2019 in Jinling Hospital. | 61 (27.0) | 165 (73.0) |
| #4 | **Internal cohort 4** | SOMATOM Definition and SOMATOM Definition Flash | 374 | Consecutive eligible CTA cases for simulated real-world validation from Jun. 1, 2019 to Jul. 31, 2019 in Jinling Hospital. | 53 (14.2) | 321 (85.8) |
| #5 | **Internal cohort 5** | SOMATOM Definition and SOMATOM Definition Flash | 333 | Consecutive eligible head CTA patients for suspected AIS from Jan. 1, 2019 to May. 31, and from Sep. 1, to Dec.31, 2019 in Jinling Hospital for validation of the function of confident removal aneurysm-negative cases to reduce radiologists’ workload. | 14 (4.2) | 319 (95.8) |
| #6 | **NBH cohort** | SOMATOM Definition AS+ , GE Revolution | 211 | DSA-verified consecutive eligible CTA cases from Jan. 1, 2019 to Jul. 31, 2019 in Nanjing Brain Hospital for validation of the effect different manufacturers and scan protocols. | 39 (18.5) | 172 (81.5) |
| #7 | **TJ cohort** | SOMATOM Definition Flash, GE Revolution CT, Toshiba Aquilion ONE | 147 | Consecutive eligible CTA cases between Jan. 1, 2013 and Dec. 31, 2018 for validation of the effect of different manufacturers in Tianjin First Central Hospital. | 109 (74.1) | 38 (25.9) |
| #8 | **LYG cohort** | SOMATOM Definition and SOMATOM Definition Flash | 316 | Consecutive eligible CTA cases for simulated real-world validation from Aug. 1, 2018 to Sep. 30, 2019 in Lianyungang First People’s Hospital. | 60 (19.0) | 256 (81.0) |

CTA: computed tomography angiography; DSA, digital subtraction angiography; IA: intracranial aneurysm.

Supplementary Table 2 | Overview of misclassification errors and the possible reasons on the validation sets

**a**

| **Variable** | **Internal cohort 2, n=245** | **Internal cohort 3, n=226** | **NBH cohort, n=211** | **TJ cohort, n=147** |
| --- | --- | --- | --- | --- |
| **Number of patients with IAs, n** | 108 | 61 | 39 | 109 |
| **Missed patients with IAs, n** | 6 | 13 | 6 | 26 |
| **Missed IAs, n** | 23 | 25 | 11 | 38 |
| **Patients with false positives, n** | 47 | 38 | 43 | 43 |
| **False Positives, n** | 63 | 44 | 57 | 64 |
| **FP/case** | 0.26 | 0.19 | 0.27 | 0.44 |

**b**

| **Reasons of misclassifications of patients** | **Internal cohort 2, n=23** | **Internal cohort 3, n=25** | **NBH cohort, n=11** | **TJ cohort, n=38** |
| --- | --- | --- | --- | --- |
| **Tiny aneurysms (<3 mm), n** | 14 | 17 | 5 | 14 |
| **Small aneurysms (<5 mm), n** | 20 | 3 | 9 | 24 |
| **Uncommon morphology of IAS, n** | 3 | 4 | 1 | 2 |
| **Uncommon locations, n** | 0 | 1 | 0 | 2 |
| **Inappropriate contrast agent protocols, n** | 0 | 1 | 0 | 0 |
| **Overshooting of bone subtraction** | 3 | 2 | 3 | 3 |
| **Other unexplained reasons, n** | 2 | 1 | 2 | 17 |

**a,** Overview of the number of misclassification errors in Internal cohort 2, Internal cohort 3, NBH cohort and TJ cohort. **b,** Overview of the possible reasons for misclassifications of the wrong cases. In general, tiny aneurysms were easily missed by the model.

FP=False positive

Supplementary Table 3 | Comparison of the performance of the model and the radiologists/ neurosurgeons in Internal cohort 4 and LYG cohort.

**a**

| **Internal cohort 4** | | **Patient-level sensitivity** | | **Specificity** | **ACC** | **PPV** | **NPV** | **Lesion-level sensitivity** | |
| --- | --- | --- | --- | --- | --- | --- | --- | --- | --- |
| **Entire** | Radiologists | 58.5%  (53.0%-63.8%) | | 95.3%  (94.2%-96.1%) | 90.1%  (88.7%-91.2%) | 67.1%  (61.4%-72.4%) | 93.3%  (92.1%-94.3%) | 50.3%  (45.5%-55.0%) | |
|  | Model | 73.6%  (59.4%-84.3%) | | 87.9%  (83.6%-91.1%) | 85.8%  (81.8%-89.1%) | 50.0%  (39.2%-60.8%) | 95.3%  (92%-97.3%) | 60.6%  (48.2%-71.7%) | |
|  | Δ(%) | 15.1%  (7.8%-22.4%) | | -7.4%  (-9.2%--5.7%) | -4.3%  (-6.2%--2.3%) | -17.1%  (-29.5%--4.7%) | 2.0%  (-0.7%-4.6%) | 20.3% | |
|  | Comparison | Superiority | | No conclusion | No conclusion | No conclusion | Noninferiority | - | |
|  | *P*^#^ | ***0.037*** | | ***<0.001*** | ***0.014*** | ***0.005*** | 0.370 | 0.107 | |
| **SAH** | Radiologists | 66.7%  (54.1%-77.3%) | | 95.4%  (89.6%-98.0%) | 85.1%  (79.0%-89.7%) | 88.9%  (76.5%-95.2%) | 83.7%  (76.2%-89.2%) | 54.8%  (44.1%-65.0%) | |
|  | Model | 80.0%  (44.2%-96.5%) | | 88.9%  (63.9%-98.1%) | 85.7%  (66.4%-95.3%) | 80.0%  (44.2%-96.5%) | 88.9%  (63.9%-98.1%) | 64.3%  (35.6%-86%) | |
|  | Δ(%) | 13.3%  (-2.3%-29%) | | -6.5%  (-13.6%-0.6%) | 0.6%  (-7%-8.1%) | -8.9%  (-35.3%-17.5%) | 5.1%  (-10.8%-21.1%) | 9.5% | |
|  | Comparison | Noninferiority | | No conclusion | No conclusion | No conclusion | No conclusion | - | |
|  | *P*^#^ | 0.636 | | 0.578 | >0.999 | 0.812 | 0.930 | 0.506 | |
| **non-SAH** | Radiologists | 56.6%  (50.5%-62.5%) | | 95.3%  (94.2%-96.2%) | 90.5%  (89.1%-91.7%) | 56.9%  (42.2%-70.4%) | 94.0%  (90.7%-96.2%) | 49.1%  (43.9%-54.4%) | |
|  | *P** | 0.154 | | 0.962 | ***0.026*** | ***<0.001*** | ***<0.001*** | 0.354 | |
|  | Model | 72.1%  (56.1%-84.2%) | | 87.8%  (83.4%-91.2%) | 85.8%  (81.6%-89.2%) | 45.6%  (33.6%-58.1%) | 95.7%  (92.4%-97.6%) | 59.6%  (45.8%-72.2%)s | |
|  | *P** | 0.910 | | >0.999 | >0.999 | ***0.042*** | 0.457 | 0.750 | |
|  | Δ(%) | 15.5%  (7.3%-23.7%) | | -7.5%  (-9.3%--5.7%) | -4.6%  (-6.6%--2.7%) | -17.3%  (-30.7%--4%) | 1.8%  (-0.9%-4.4%) | 10.5% | |
|  | Comparison | Superiority | | No conclusion | No conclusion | No conclusion | Noninferiority | - | |
|  | *P*^#^ | 0.056 | | ***<0.001*** | ***0.009*** | ***0.011*** | 0.244 | 0.141 | |
| **b** |  |  | |  |  |  |  |  | |
| **Internal cohort 4** | | | **Patient-level sensitivity** | **Specificity** | **ACC** | **PPV** | **NPV** | **Lesion-level sensitivity** |  |
| **Entire** | neurosurgeons | | 66.0%  (56.1%-74.8%) | 93.9%  (91.7%-95.6%) | 90.0%  (87.5%-92.0%) | 64.2%  (54.4%-73.0%) | 94.4%  (92.2%-96.0%) | 54.2%  (45.7%-62.5%) |  |
|  | Model | | 73.6%  (59.4%-84.3%) | 87.9%  (83.6%-91.1%) | 85.8%  (81.8%-89.1%) | 50.0%  (39.2%-60.8%) | 95.3%  (92%-97.3%) | 60.6%  (48.2%-71.7%) |  |
|  | Δ(%) | | 7.5%  (-4.8%-19.9%) | -6.1%  (-9.2%--2.9%) | -4.1%  (-7.4%--0.8%) | -14.2%  (-28.5%-0.1%) | 0.9%  (-2.1%-3.9%) | 6.4% |  |
|  | Comparison | | Noninferiority | No conclusion | No conclusion | No conclusion | Noninferiority | ***-*** |  |
|  | *P*^#^ | | 0.334 | ***0.001*** | ***0.040*** | 0.052 | 0.568 | 0.379 |  |
| **SAH** | neurosurgeons | | 80.0%  (55.7%-93.4%) | 83.3%  (66.5%-93.0%) | 82.1%  (69.2%-90.7%) | 72.7%  (49.6%-88.4%) | 88.2%  (71.6%-96.2%) | 64.3%  (44.1%-80.7%) |  |
|  | Model | | 80.0%  (44.2%-96.5%) | 88.9%  (63.9%-98.1%) | 85.7%  (66.4%-95.3%) | 80.0%  (44.2%-96.5%) | 88.9%  (63.9%-98.1%) | 64.3%  (35.6%-86%) |  |
|  | Δ(%) | | 0%  (-24.8%-24.8%) | 5.6%  (-10.4%-21.5%) | 3.6%  (-10%-17.2%) | 7.3%  (-23.7%-38.3%) | 0.7%  (-17.5%-18.8%) | 0% |  |
|  | Comparison | | No conclusion | No conclusion | No conclusion | No conclusion | No conclusion | - |  |
|  | *P*^#^ | | >0.999 | 0.892 | 0.918 | >0.999 | >0.999 | >0.999 |  |
| **non-SAH** | neurosurgeons | | 62.8%  (51.6%-72.8%) | 94.6%  (92.4%-96.2%) | 90.6%  (88.1%-92.6%) | 62.1%  (51%-72.1%) | 94.7%  (92.5%-96.3%) | 51.8%  (42.2%-61.1%) |  |
|  | *P** | | 0.143 | ***0.017*** | ***0.043*** | 0.351 | 0.226 | 0.233 |  |
|  | Model | | 72.1%  (56.1%-84.2%) | 87.8%  (83.4%-91.2%) | 85.8%  (81.6%-89.2%) | 45.6%  (33.6%-58.1%) | 95.7%  (92.4%-97.6%) | 59.6%  (45.8%-72.2%)s |  |
|  | *P** | | 0.910 | >0.999 | >0.999 | ***0.042*** | 0.457 | 0.750 |  |
|  | Δ(%) | | 9.3%  (-4.6%-23.2%) | -6.8%  (-9.9%--3.6%) | -4.8%  (-8.2%--1.4%) | -16.5%  (-32.1%--0.9%) | 1.0%  (-2.0%-4.0%) | 7.8% |  |
|  | Comparison | | Noninferiority | No conclusion | No conclusion | No conclusion | Noninferiority | - |  |
|  | *P*^#^ | | 0.293 | ***<0.001*** | ***0.021*** | ***0.041*** | 0.537 | 0.329 |  |

**c**

| **LYG cohort** | | **Patient-level sensitivity** | | **Specificity** | **ACC** | **PPV** | **NPV** | **Lesion-level sensitivity** | |
| --- | --- | --- | --- | --- | --- | --- | --- | --- | --- |
| **Entire** | Radiologists | 70.8%  (65.9%-75.3%) | | 95.6%  (94.4%-96.5%) | 90.9%  (89.5%-92.1%) | 78.9%  (74.2%-83.0%) | 93.3%  (92.0%-94.5%) | 61.6%  (57.1%-66.0%) | |
|  | Model | 85.0%  (72.9%-92.5%) | | 74.6%  (68.7%-79.7%) | 76.6%  (71.4%-81.1%) | 44.0%  (34.9%-53.5%) | 95.4%  (91.4%-97.8%) | 78.9%  (67.8%-87.1%) | |
|  | Δ(%) | 14.2%  (8.2%-20.1%) | | -21%  (-23.4%--18.6%) | -14.3%  (-16.6%--12%) | -35%  (-45%--24.9%) | 2.2%  (-1%-5.3%) | 17.3% | |
|  | Comparison | Superiority | | No conclusion | No conclusion | No conclusion | Noninferiority | ***-*** | |
|  | *P*^#^ | ***0.022*** | | ***<0.001*** | ***<0.001*** | ***<0.001*** | 0.237 | 0.004 | |
| **SAH** | Radiologists | 81.3%  (74.3%-86.8%) | | 96.2%  (91.4%-98.4%) | 88.3%  (84.0%-91.5%) | 96.1%  (91.1%-98.3%) | 82.5%  (63.9%-92.6%) | 72.4%  (65.7%-78.2%) | |
|  | Model | 92.0%  (72.5%-98.6%) | | 72.7%  (49.6%-88.4%) | 83.0%  (68.7%-91.9%) | 79.3%  (59.7%-91.3%) | 88.9%  (63.9%-98.1%) | 87.5%  (70.1%-95.9%) | |
|  | Δ(%) | 10.7%  (3.1%-18.3%) | | -23.5%  (-31.8%--15.2%) | -5.7%  (-11.4%-0.1%) | -16.8%  (-31.9%--1.7%) | 6.4%  (-9.3%-22.1%) | 15.1% | |
|  | Comparison | Noninferiority | | No conclusion | No conclusion | No conclusion | No conclusion | - | |
|  | *P*^#^ | 0.306 | | ***<0.001*** | 0.307 | ***0.005*** | 0.683 | 0.069 | |
| **non-SAH** | Radiologists | 63.3%  (56.6%-69.6%) | | 95.5%  (94.3%-96.5%) | 91.3%  (89.9%-92.6%) | 67.7%  (50.5%-81.1%) | 94.5%  (90.8%-96.8%) | 53.8%  (47.8%-59.7%) | |
|  | *P** | ***<0.001*** | | 0.709 | ***<0.001*** | ***<0.001*** | ***<0.001*** | ***<0.001*** | |
|  | Model | 80.0%  (62.5%-90.9%) | | 74.8%  (68.6%-80.1%) | 75.5%  (69.8%-80.4%) | 32.2%  (22.8%-43.2%) | 96.2%  (91.9%-98.3%) | 72.7%  (57%-84.5%) | |
|  | *P** | 0.359 | | 0.832 | 0.262 | ***<0.001*** | 0.411 | 0.119 | |
|  | Δ(%) | 16.7%  (3.1%-18.3%) | | -20.7%  (-23.2%--18.2%) | -15.8%  (-18.3%--13.3%) | -35.5%  (-47.3%--23.7%) | 1.7%  (-1.4%-4.7%) | 18.9% | |
|  | Comparison | Noninferiority | | No conclusion | No conclusion | No conclusion | Noninferiority | - | |
|  | *P*^#^ | 0.054 | | ***<0.001*** | ***<0.001*** | ***<0.001*** | 0.367 | ***0.019*** | |
| **d** |  |  | |  |  |  |  |  | |
| **LYG cohort** | | | **Patient-level sensitivity** | **Specificity** | **ACC** | **PPV** | **NPV** | **Lesion-level sensitivity** |  |
| **Entire** | Neurosurgeons | | 70.8%  (61.7%-78.6%) | 95.1%  (92.8%-96.8%) | 90.5%  (87.9%-92.6%) | 77.3%  (68.1%-84.5%) | 93.3%  (90.7%-95.2%) | 74.3%  (66.5%-80.9%) |  |
|  | Model | | 85.0%  (72.9%-92.5%) | 74.6%  (68.7%-79.7%) | 76.6%  (71.4%-81.1%) | 44.0%  (34.9%-53.5%) | 95.4%  (91.4%-97.8%) | 78.9%  (67.8%-87.1%) |  |
|  | Δ(%) | | 14.2%  (3.8%-24.5%) | -20.5%  (-24.7%--16.3%) | -13.9%  (-17.9%--9.9%) | -33.3%  (-45.3%--21.4%) | 2.2%  (-1.4%-5.8%) | 4.6% |  |
|  | Comparison | | Noninferiority | No conclusion | No conclusion | No conclusion | Noninferiority | ***-*** |  |
|  | *P*^#^ | | ***0.037*** | ***<0.001*** | ***<0.001*** | ***<0.001*** | 0.268 | 0.444 |  |
| **SAH** | Neurosurgeons | | 78.0%  (63.7%-88.0%) | 95.5%  (83.3%-99.2%) | 86.2%  (77.2%-92.1%) | 95.1%  (82.2%-99.2%) | 79.2%  (65.5%-88.7%) | 76.6%  (64%-85.9%) |  |
|  | Model | | 92.0%  (72.5%-98.6%) | 72.7%  (49.6%-88.4%) | 83.0%  (68.7%-91.9%) | 79.3%  (59.7%-91.3%) | 88.9%  (63.9%-98.1%) | 87.5%  (70.1%-95.9%) |  |
|  | Δ(%) | | 14%  (0.3%-27.7%) | -22.7%  (-37.3%--8.2%) | -3.2%  (-13.5%-7.1%) | -15.8%  (-32%-0.3%) | 9.6%  (-8.5%-27.8%) | 10.9% |  |
|  | Comparison | | Noninferiority | No conclusion | No conclusion | No conclusion | No conclusion | - |  |
|  | *P*^#^ | | 0.235 | ***0.023*** | 0.616 | 0.096 | 0.575 | ***<0.001*** |  |
| **non-SAH** | Neurosurgeons | | 65.7%  (53.3%-76.4%) | 95.1%  (92.6%-96.8%) | 91.3%  (88.5%-93.4%) | 66.7%  (54.2%-77.3%) | 94.9%  (92.4%-96.6%) | 72.7%  (62%-81.4%) |  |
|  | *P** | | 0.144 | >0.999 | 0.120 | ***<0.001*** | ***<0.001*** | 0.593 |  |
|  | Model | | 80.0%  (62.5%-90.9%) | 74.8%  (68.6%-80.1%) | 75.5%  (69.8%-80.4%) | 32.2%  (22.8%-43.2%) | 96.2%  (91.9%-98.3%) | 72.7%  (57%-84.5%) |  |
|  | *P** | | 0.359 | 0.832 | 0.262 | ***<0.001*** | 0.411 | 0.119 |  |
|  | Δ(%) | | 14.3%  (-0.3%-28.8%) | -20.3%  (-24.7%--15.9%) | -15.8%  (-20.1%--11.5%) | -34.5%  (-49.3%--19.6%) | 1.3%  (-2.2%-4.7%) | 0.0% |  |
|  | Comparison | | Noninferiority | No conclusion | No conclusion | No conclusion | Noninferiority | - |  |
|  | *P*^#^ | | 0.130 | ***<0.001*** | ***<0.001*** | ***<0.001*** | 0.494 | >0.999 |  |

**a, c,** Comparison of the performance of the model and the radiologists and neurosurgeons in Internal cohort 4. **b, d,** Comparison of the performance of the model and the radiologists and neurosurgeon in LYG cohort.

Superiority comparisons on the Internal cohort 4 and LYG cohort data were conducted using Obuchowski’s extension of the two-sided McNemar test for clustered data. Non-inferiority comparisons were Wald tests using the Obuchowski correction. Comparisons were performed with a two-sided permutation test. The data in parentheses are 95% confidence interval.

*P* ^#^: indicates difference of performance between radiologists/neurosurgeons and the model.

*P**: indicates difference of performance of radiologists/neurosurgeons and that of the model between SAH group and non-SAH group.

SAH, subarachnoid hemorrhage; ACC, accuracy; NPV, negative predictive value; PPV, positive predictive value.

Note: the performances of radiologists were calculated as the micro-average metrics of every radiologists/neurosurgeons. The validated results at *P*<0.05 are in bold and italic.

Supplementary Table 4 | Comparison of the performances of the model and the most frequently employed 3D U-net model using the same training data (Internal cohort 1)

| **Model** | **Accuracy** | **Patient-level sensitivity** | **Specificity** | **PPV** | **NPV** | **Lesion-level sensitivity** | **Dice** |
| --- | --- | --- | --- | --- | --- | --- | --- |
| **U-Net_3D** | 73.3%  (65.7%-79.8%) | 94.7%  (87.1%-97.9%) | 52.0%  (40.9%-62.9%) | 66.4%  (57.0%-74.6%) | 90.7%  (78.4%-96.3%) | 92.2%  (84.8%-96.2%) | 0.666  (0.611-0.721) |
| **DAResU-Net** | 86.0%  (79.5%-90.7%) | 97.3%  (90.8%-99.3%) | 74.7%  (63.8%-83.1%) | 79.4%  (70.0%-86.4%) | 96.6%  (88.3%-99.0%) | 95.6%  (89.1%-98.3%) | 0.752  (0.708-0.796) |
| ***p*** | ***0.006*** | 0.405 | ***0.004*** | ***0.041*** | 0.421 | 0.351 | ***0.006*** |

The data in parentheses are 95% confidence interval.

NPV, negative predictive value; PPV, positive predictive value. The validated results at *p*<0.05 are in bold and italic.
